# Carbapenem-resistant Enterobacteriaceae colonization (CRE) and subsequent risk of infection and 90-day mortality in critically ill patients, an observational study

**DOI:** 10.1371/journal.pone.0186195

**Published:** 2017-10-12

**Authors:** Thomas Howe McConville, Sean Berger Sullivan, Angela Gomez-Simmonds, Susan Whittier, Anne-Catrin Uhlemann

**Affiliations:** 1 Department of Medicine, Division of Infectious Diseases, Columbia University Medical Center, New York, New York, United States of America; 2 Department of Pathology and Cell Biology, Clinical Microbiology Laboratory, Columbia University Medical Center, New York, New York, United States of America; Azienda Ospedaliero Universitaria Careggi, ITALY

## Abstract

**Background:**

Carbapenem-resistant Enterobacteriaceae (CRE) have emerged as an urgent public health threat. Intestinal colonization with CRE has been identified as a risk factor for the development of systemic CRE infection, but has not been compared to colonization with third and/or fourth generation cephalosporin-resistant (Ceph-R) Enterobacteriaceae. Moreover, the risk conferred by colonization on adverse outcomes is less clear, particularly in critically ill patients admitted to the intensive care unit (ICU).

**Methods:**

We carried out a cohort study of consecutive adult patients screened for rectal colonization with CRE or Ceph-R upon ICU entry between April and July 2013. We identified clinical variables and assessed the relationship between CRE or Ceph-R colonization and subsequent systemic CRE infection within 30 days (primary outcome) and all-cause mortality within 90 days (secondary outcome).

**Results:**

Among 338 ICU patients, 94 (28%) were colonized with either Ceph-R or CRE. 26 patients developed CRE infection within 30 days of swab collection; 47% (N = 17/36) of CRE-colonized and 3% (N = 2/58) of Ceph-R colonized patients. 36% (N = 13/36) of CRE-colonized patients died within 90 days compared to 31% (N = 18/58) of Ceph-R-colonized and 15% (N = 37/244) of non-colonized patients. In a multivariable analysis, CRE colonization independently predicted development of a systemic CRE infection at 30 days (aOR 10.8, 95% CI2.8–41.9, p = 0.0006); Ceph-R colonization did not (aOR 0.5, 95% CI0.1–3.3, p = 0.5). CRE colonization was associated with increased 90-day mortality in a univariable analysis (p-value 0.001), in a multivariable model, previous hospitalization and medical ICU admission were independent predictors of 90-day mortality whereas CRE colonization approached significance (aOR 2.3, 95% CI1.0–5.3, p = 0.056).

**Conclusions:**

Our study highlights the increased risk of CRE infection and mortality in patients with CRE colonization at the time of ICU admission. Future studies are needed to assess how CRE colonization can guide empiric antibiotic choices and to develop novel decolonization strategies.

## Introduction

Carbapenem-resistant Enterobacteriaceae (CRE) have become widespread in the US, prompting the Centers for Diseases Control and Prevention (CDC) to classify them as an urgent threat to public health, its highest risk category [1]. CRE infections account for an estimated 140,000 cases of healthcare-associated infections annually [1], and produce high attributable mortality rates of 26–44% [2]. Infections with CRE disproportionately affect severely ill patients with multiple comorbidities [3]. Patients residing in the intensive care unit (ICU) have been found to have a particularly high burden of CRE infections as well as increased mortality [4–6]. Immunocompromised patients, organ transplant recipients, and patients with previous antibiotic exposure and central venous catheters are also at increased risk of infection with CRE [7–9].

The majority of CRE infections worldwide are caused by *K*. *pneumoniae*. Recently, carbapenem resistance has also been increasingly reported in *Escherichia coli*, *Klebsiella oxytoca*, and *Enterobacter cloacae* [10, 11]. One of the potential endogenous reservoirs of CRE is the human intestine, where Enterobacteriaceae, including *K*. *pneumoniae*, can reside as colonizers. In endemic areas, the prevalence of CRE colonization in hospitalized patients ranges from 3–7% [12–17], but can be higher in patients admitted to intensive care units (ICU) [5]. Colonization with CRE is a known risk factor for subsequent CRE infections. In a recent meta-analysis of 1,806 patients colonized with CRE, the overall risk of systemic CRE infection was 16.5% [6]. In ICU patients, this risk varied widely and between 29–73% of carbapenem-resistant *K*. *pneumoniae* carriers subsequently developed infections [4, 5]. Mortality in ICU CRE carriers was high and ranged between 26 to 41% [5, 18, 19]. However, variability in CRE organisms, study population and study design (e.g. outbreak versus endemic setting) have allowed limited conclusions about the clinical importance and utility of identifying CRE carriers. Screening for resistant Gram-negative (CRE, Ceph-R) intestinal carriage is not the universal standard of care, although some experts advocate surveillance during outbreaks as part of infection control initiatives [10, 20]. While Ceph-R colonization has similar risk factors and is associated with increased use of broad-spectrum antibiotics [21] the role of colonization with third and/or fourth generation cephalosporin-resistant Enterobacteriaceae (Ceph-R) on subsequent CRE infections is less clear.

As part of a non-outbreak surveillance initiative at our institution located in an area of high CRE endemicity, rectal screening was performed in medical and surgical ICUs to identify carriage with CRE Ceph-R. Here, we examined the rates of fecal colonization with CRE and/or Ceph-R at the time of ICU admission and determined the risk of 30-day CRE infection (primary outcome) and all-cause mortality at 90 days post ICU admission (secondary outcome).

## Materials and methods

### Study population

This was an observational cohort study performed at a large academic medical center and an affiliated community hospital in Northern Manhattan. The Columbia University Medical Center (CUMC) institutional review board approved this study (IRB AAAM 6617) and waived the need for informed consent. We included all patients >18 years old who had undergone evaluation for rectal colonization with multi-drug resistant (MDR) Gram-negative organisms on admission to one of the adult medical ICUs (MICU) or surgical ICUs (SICU) between April 7^th^ 2013 to July 8^th^ 2013. During this 3-month period rectal swabs (BD CultureSwab, Becton, Dickinson) from 338 patients collected at the time of admission to the four intensive care units at our center were processed by the clinical microbiology laboratory. Collected samples were streaked onto Remel Spectra Chromogenic Agar (Remel). Positive colonies were tested for species identification and drug susceptibilities using VITEK according to the CLSI guidelines [22]. CRE were designated on the basis of meropenem minimum inhibitory concentration (MIC) ≥ 2 μg/ml or ertapenem MIC ≥1 μg/ml. Gram-negative bacteria not belonging to family Enterobacteriaceae were also identified using this approach. Isolates that were carbapenem susceptible but exhibited third and/or fourth generation cephalosporin resistance, were designated Ceph-R.

We extracted demographic and clinical information from the patient’s electronic charts, including ICU admission diagnosis, prior hospital admissions or transfer from a nursing home/long-term care facility over the past 6 months, and antibiotic exposure in the inpatient setting within the past 6 months. The Charlson Comorbidity Index score (CCIS) was calculated from clinical data as a marker of patients’ underlying health [23]. Charts were also reviewed for both previous positive cultures with CRE over the 6 months prior to ICU admission and for subsequent development of infections with these organisms for up to 90 days following ICU admission and swab collection. Previous positive cultures represented blood, respiratory, wound, or urine cultures positive for CRE. Subsequent infections were classified based on positive clinical cultures and meeting NHSN criteria for infections [24]. All patients were assumed to receive standard ICU level care, including, but not limited to standard isolation precautions and ventilator bundles when intubated. Notably, Surveillance results were not available to the treating physicians at the time of culture and thus did not factor into clinical decisions including isolation precautions and antibiotic management.

### Statistical analysis

Patients were categorized according to results of screening for rectal colonization on ICU admission as CRE-colonized, Ceph-R-colonized or non-colonized. Clinical parameters were described as frequencies or using the median and interquartile range (IQR). In an analysis of factors associated with primary and secondary outcomes, variables between groups were compared using χ^2^ or Fisher’s exact test for categorical variables and unpaired t-test or Mann-Whitney-Wilcoxon test for continuous variables as appropriate. Variables with a p-value <0.05 were considered for inclusion in a multivariable logistic model. Variables that were strongly correlated with variables already included in the model were left out of the analysis (i.e. admission diagnosis and ICU type). Other variables (i.e. gender, age, CCIS, and ICU type) were included *a priori* as they were considered potential confounders. As part of the multivariable logistic model for 90 day mortality a mediation analysis was performed for CRE infection given the causal hypothesis that CRE colonization increases risk for subsequent CRE infection and as a result mortality.

The primary outcome measure in this study was the development of CRE infection within 30 days of the positive swab. Our secondary outcome measure was 90-day mortality from the date of swab collection. Additional analyses were performed using survival analysis methods. Specifically, the relationship between CRE and/or Ceph-R colonization and both the primary and secondary outcomes was analyzed using Kaplan-Meier curves and the log-rank test. In the analysis of infection within 30 days, patients who died were censored. Data were analysed using SAS 9.4 (SAS Institute Inc., Cary, NC).

## Results

### Clinical characteristics of ICU patient cohort

The study population encompassed 103 SICU and 235 MICU patients who were screened for fecal colonization upon entry to the ICU. Study participants (n = 338) were predominantly male (n = 184, 54%) with an average age of 63 years (range 19–95) at the time of ICU admission (Table 1). Thirty-six patients (11%) were colonized with CRE. The majority of these were accounted for by *K*. *pneumoniae* (n = 33, 92%, Table 1). The CRE screening test also revealed the presence of colonization with Ceph-R *Enterobacteriaceae* in 58 patients (Table 1). The majority of Ceph-R isolates were *E*. *coli* (n = 39, 67%) followed by *K*. *pneumoniae* (n = 9, 16%).

**Table 1:** Baseline characteristics of intensive care unit study population.

Variable n (%)	Colonized with Ceph-R (n = 58)	Colonized with CRE (n = 36)		Any colonization (n = 94)		Non-colonized (n = 244)		2-group p-value*	3-group p-value**	
**Clinical characteristics**										
**Male sex**	30 (52%)	21 (58%)		51 (54%)		133 (55%)		1.0	0.1	
**Age > 65 years**	32 (55%)	18 (50%)		50 (53%)		112 (46%)		0.2	0.4	
**Admitted From**								0.0002	<0.0001	
Home	42 (72%)	20 (56%)		62 (66%)		209 (86%)				
SNF or Group Home	9 (16%)	14 (39%)		23 (24%)		22 (9%)				
Outside Hospital	7 (12%)	2 (6%)		9 (10%)		13 (5%)				
**ICU Type**								0.3	0.2	
Surgical	12 (21%)	13 (36%)		25 (27%)		78 (32%)				
Medical	46 (79%)	23 (64%)		69 (73%)		166 (68%)				
**ICU Admission Diagnosis**								0.2	0.5	
Surgery	13 (22%)	10 (28%)		23 (24%)		83 (34%)				
Shock	20 (34%)	13 (36%)		33 (35%)		68 (28%)				
Respiratory Failure	18 (31%)	8 (22%)		26 (28%)		53 (22%)				
Metabolic Disarray	5 (9%)	3 (8%)		8 (9%)		18 (7%)				
Altered Mental Status	2 (3%)	2 (6%)		4 (4%)		22 (9%)				
**CCIS (mean, IQR)**	3 (2,4)	3 (2,4)		3 (2,4)		3 (1,4)		0.2	0.4	
Type II DM	21 (36%)	7 (19%)		28 (30%)		79 (32%)		0.7	0.2	
Heart Disease	11 (19%)	1 (3%)		12 (13%)		18 (7%)		0.1	0.008	
Pulmonary Disease	18 (31%)	15 (42%)		33 (35%)		66 (27%)		0.14	0.2	
Liver Disease	10 (17%)	14 (39%)		24 (36%)		34 (14%)		0.01	0.001	
Malignancy	11 (19%)	5 (14%)		16 (17%)		65 (27%)		0.06	0.2	
Renal Disease	13 (22%)	6 (17%)		19 (20%)		48 (20%)		0.9	0.8	
**Previous SOT**	13 (22%)	9 (25%)		22 (23%)		22 (9%)		0.0004	0.002	
**Within the previous 6 months**										
**Hospitalization**	39 (67%)	30 (83%)		69 (73%)		106 (43%)		<0.0001	<0.0001	
**Endoscopies**										
EGD or colonoscopy	10 (17%)	11 (31%)		21 (22%)		32 (13%)		0.04	0.03	
ERCP or EUS	2 (3%)	2 (6%)		4 (4%)		4 (2%)		0.2	0.1	
**Antibiotic Exposure**	56 (97%)	36 (100%)		92 (98%)		174 (71%)		<0.0001	<0.0001	
Piperacillin/Tazobactam	34 (59%)	28 (78%)		62 (66%)		108 (44%)		0.0004	0.0003	
Carbapenem	11 (19%)	11 (31%)		22 (23%)		11 (5%)		<0.0001	<0.0001	
1^st^ or 2^nd^-generation cephalosporin	21 (36%)	16 (44%)		37 (39%)		77 (32%)		0.2	0.3	
3rd or 4^th^-generation Cephalosporin	17 (29%)	14 (39%)		31 (33%)		54 (22%)		0.04	0.07	
Fluoroquinolone	13 (22%)	13 (36%)		26 (28%)		42 (17%)		0.03	0.03	
Aminoglycoside	18 (31%)	13 (36%)		31 (33%)		40 (16%)		0.0008	0.003	
Vancomycin	34 (59%)	26 (72%)		60 (64%)		87 (36%)		<0.0001	<0.0001	
TMP/SMX	6 (10%)	2 (6%)		8 (9%)		12 (5%)		0.8	0.3	
Tetracycline	2 (3%)	5 (14%)		7 (7%)		3 (1%)		0.001	0.001	
Polymyxin	2 (3%)	4 (11%)		6 (6%)		3 (1%)		0.02	0.005	
Macrolide	17 (29%)	12 (33%)		29 (31%)		51 (21%)		0.05	0.1	
**Previous infection**										
Ceph-R or CRE infection	21 (36%)	22 (61%)		43 (46%)		15 (6%)		<0.0001	<0.0001	
Ceph-R infection	22 (38%)	9 (25%)		31 (33%)		13 (5%)		<0.0001	<0.0001	
CRE infection	3 (5%)	19 (53%)		22(23%)		2 (1%)		<0.0001	<0.0001	
**Colonizing organism**										
*E*. *coli*	39 (67%)	0 (0%)		39 (41%)						
*K*. *pneumoniae*	9 (16%)	33 (92%)		42 (45%)						
Other	10 (17%)	3 (8%)		13 (14%)						

*p-value reflects a 2-way comparison between either Ceph-R- or CRE-colonized (i.e. Any colonization) and non-colonized patients

** p-value reflects a 3-way comparison between Ceph-R-colonized, CRE-colonized, and non-colonized patients

Abbreviations: Ceph-R, 3^rd^/4^th^ generation cephalosporin-resistant Enterobacteriaceae; CRE, carbapenem-resistant Enterobacteriaceae; SNF, skilled nursing facility; ICU, intensive care unit; CCIS, Charlson Comorbidity Index score; IQR, intraquartile range; DM, diabetes mellitus; SOT, solid organ transplant; EGD, esophagogastroduodenoscopy; ERCP, endoscopic retrograde cholangiopancreatography; EUS, endoscopic ultrasound; TMP/SMX, trimethoprim/sulfamethoxazole.

Characteristics of CRE-colonized, Ceph-R-colonized, and non-colonized patients are summarized in Table 1. Patients colonized with either CRE and/or Ceph-R were significantly more likely than non-colonized patients to have a recent hospitalization or subacute nursing facility (SNF) admission, underlying liver disease, history of solid organ transplantation (SOT), and antibiotic exposure within the past 6 months, except to 1^st^ or 2^nd^ generation cephalosporins, trimethoprim-sulfamethoxazole and macrolides (Table 1). Overall, colonized and non-colonized groups did not significantly differ by age, gender, ICU admission diagnosis including septic shock, CCIS or ICU location (Table 1).

CRE-colonized patients had significantly higher rates of liver disease compared to non-colonized patients (Bonferroni-corrected p = 0.0006). Ceph-R-colonized patients more often had heart disease compared to non-colonized patients (Bonferroni-corrected p = 0.02; Table 1). All CRE-colonized patients and 97% of Ceph-R-colonized patients had received antibiotic therapy within the past 6 months, compared to 71% of non-CRE colonized patients (Table 1). This included exposure to carbapenems in nearly one third of the CRE-colonized group. It is notable that in the 6 months prior to swab collection, 19 (53%) CRE-colonized patients had a previous CRE infection and 9 (25%) were infected with a Ceph-R (Table 1). Ceph-R-colonized patients had a similarly high rate of previous Ceph-R infections (n = 22, 38%). Among Ceph-R and non-colonized patients, previous CRE infections were uncommon (n = 3, 5%; n = 2, 1%; respectively).

### Characteristics of CRE infections

CRE infections developed in 26 patients (8%) within 30 days and 32 (9%) patients within 90 days. Pneumonia was the most common type of infection occurring within 30 days (n = 23, 88%), followed by urinary tract infections (n = 5, 19%). Four patients developed bloodstream infections (15%) and two had wound and intra-abdominal infections (8%). There were 9 patients with infections at multiple sites (35%).

Of 36 CRE-colonized patients, 17 (47%) developed a CRE infection within 30 days and 19 (53%) within 90 days of swab collection. Seven patients (19%) who were CRE-colonized subsequently developed infection with a Ceph-R organism. In Ceph-R-colonized patients, Ceph-R infection occurred in 13 (22%) patients within 30 days of swab collection, which was higher than the number of infections seen in the non-colonized group (n = 9, 4%), but comparable to the CRE colonized group (n = 7, 19%). In contrast, colonization with Ceph-R was not associated with subsequent CRE infection.

**Fig 1:**
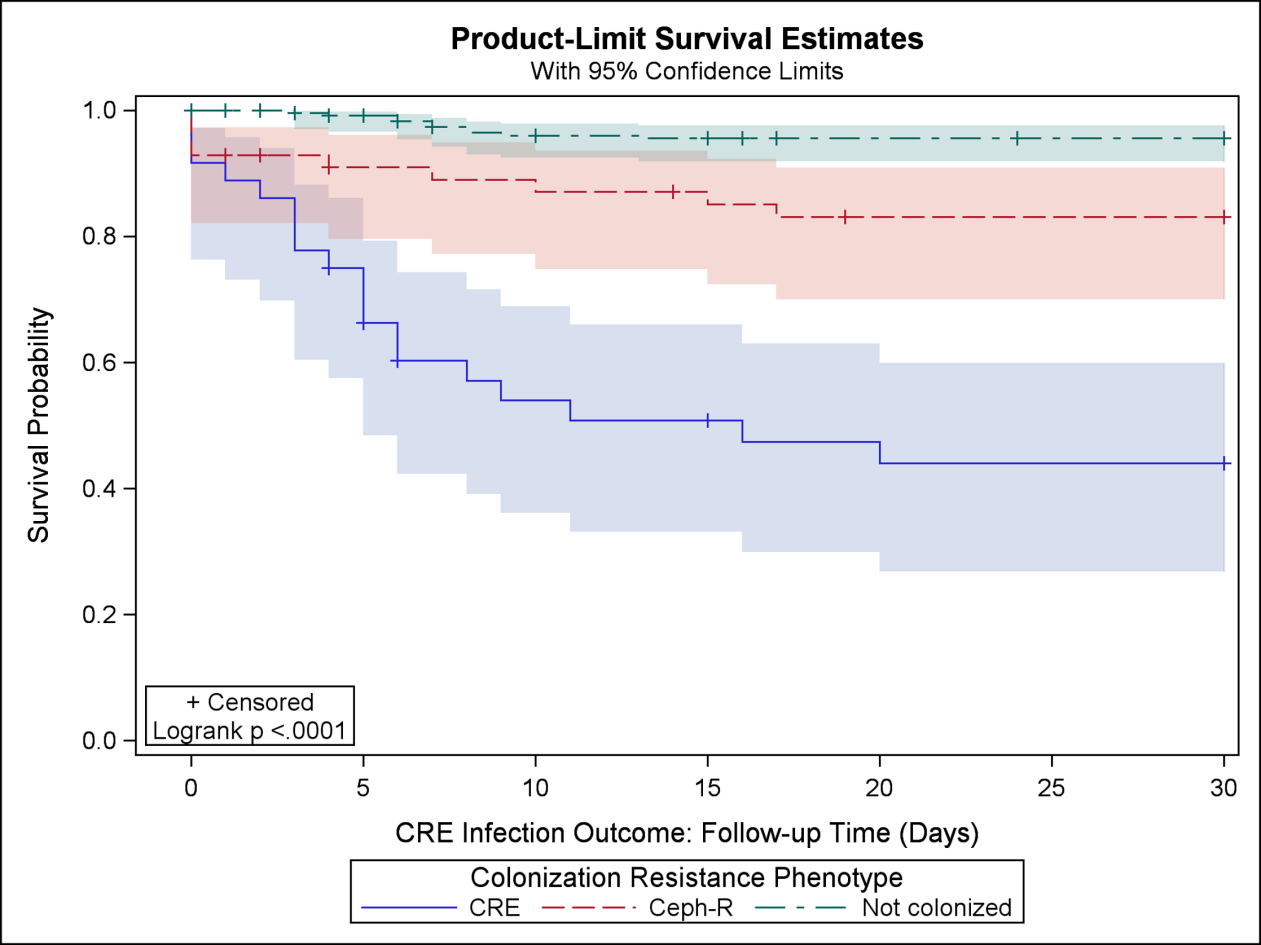
Kaplan-Meier estimates (and 95% confidence limits) of the probability of remaining uninfected with CRE, 30 day follow up.

In univariable analysis, colonization status was significantly associated with development of subsequent CRE infection. These infections occurred much more commonly in patients with antecedent CRE colonization (n = 17, 65%) than in Ceph-R colonized (n = 2, 8%) and non-colonized patients (n = 7, 27%, p<0.0001). This association held when analyzed using Kaplan-Meier estimates of the probability of remaining uninfected with CRE (Fig 1, log-rank p <0.0001). CRE infected patients were also more likely than uninfected patients to have been recently admitted to a hospital or SNF, undergone an endoscopic procedure, or taken antibiotics within the previous 6 months; these patients also had higher rates of liver disease (Table 2). Patients with CRE infections compared to uninfected patients also differed in prior infection status (overall p<0.0001), as they were more likely to have a history of prior infection with carbapenem-resistant (n = 12, 46% versus n = 11, 4%) and Ceph-R organisms (n = 4, 15% versus n = 31, 10%).

**Table 2:** Predictors of CRE infection within 30 days of intensive care unit admission.

Variable n (%)	No infection (n = 312)	Infection (n = 26)	Univariable p-value	Adjusted OR (95% CI)	Multivariable p-value
**Male sex**	169 (54%)	15 (58%)	0.7	0.8 (0.3, 2.5)	0.8
**Age > 65 years**	152 (49%)	10 (38%)	0.3	0.6 (0.2, 1.7)	0.4
**Admitted From**			0.0005		
Home	258 (83%)	13 (50%)			
SNF or Group Home	36 (12%)	9 (35%)			
Outside Hospital	18 (6%)	4 (15%)			
**ICU Type**			0.7		0.9
Surgical	96 (31%)	7 (27%)		REF	
Medical	216 (69%)	19 (73%)		1.1 (0.3, 3.5)	
**ICU Admission Diagnosis**			0.04		
Surgery	101 (32%)	5 (19%)			
Shock	94 (30%)	7 (27%)			
Respiratory Failure	70 (22%)	9 (35%)			
Metabolic Disarray	21 (7%)	5 (19%)			
Altered Mental Status	26 (8%)	0 (0%)			
**CCIS (mean, IQR)**	3 (2,4)	3.2 (2,4)	0.9	0.9 (0.7, 1.2)	0.6
Type II DM	101 (32%)	6 (23%)	0.3		
Heart Disease	28 (9%)	2 (8%)	1.0		
Pulmonary Disease	88 (28%)	11 (42%)	0.1		
Liver Disease	49 (16%)	9 (35%)	0.03		
Malignancy	77 (25%)	4 (15%)	0.3		
Renal Disease	63 (20%)	4 (15%)	0.6		
**Previous SOT**	40 (13%)	4 (15%)	0.8		
**Within the previous 6 months**					
**Hospitalization**	151 (48%)	24 (92%)	<0.0001	6.6 (1.3, 34.3)	0.03
**Endoscopies**					
EGD or colonoscopy	42 (13%)	11 (42%)	0.0006	3.7 (1.2, 11.1)	0.02
ERCP or EUS	6 (2%)	2 (8%)	0.1		
**Antibiotic use**	241 (77%)	25 (96%)	0.02	1.1 (0.1, 1.5)	0.9
**Previous Ceph-R- or CRE infection**			<0.0001		0.2*
No previous Ceph-R or CRE infection	270 (87%)	10 (38%)		REF	
Previous Ceph-R infection	31 (10%)	4 (15%)		2.1 (0.5, 9.6)	0.3
Previous CRE infection	11 (4%)	12 (46%)		3.5 (0.8, 14.8)	0.08
**Colonization Resistance Phenotype**			<0.0001		0.0003*
Non-colonized	237 (76%)	7 (27%)		REF	
Ceph-R colonized	56 (18%)	2 (8%)		0.5 (0.1, 3.3)	0.5
CRE colonized	19 (6%)	17 (65%)		10.8 (2.8, 41.9)	0.0006

*Global p-values for categorical variables in the multivariable model

Abbreviations: OR, odds ratio; SNF, skilled nursing facility; ICU, intensive care unit; CCIS, Charlson Comorbidity Index score; IQR, intraquartile range; DM, diabetes mellitus; SOT, solid organ transplant; EGD, esophagogastroduodenoscopy; ERCP, endoscopic retrograde cholangiopancreatography; EUS, endoscopic ultrasound; Ceph-R, 3^rd^/4^th^ generation cephalosporin-resistant Enterobacteriaceae.

In the final multivariable model, after controlling for age, gender, CCIS, ICU type, prior upper endoscopy or colonoscopy, antibiotic use, previous CRE or Ceph-R infection and hospitalization within the past 6 months, CRE colonization compared to non-colonization was associated with a 10.8-fold increased odds of CRE infection at 30 days (95% CI 2.8–41.9, p = 0.0006; Table 2). Ceph-R colonization was not significantly associated with subsequent CRE infection. Hospitalization within the past 6 months and previous EGD or colonoscopy were also significant predictors of 30-day CRE infection in this model.

### All cause 90-day mortality after rectal swab collection

The 90-day all-cause mortality in this cohort of ICU patients was 20% (n = 68). Compared to non-colonized patients, patients colonized with either a Ceph-R or CRE had significantly higher mortality within 90 days of swab collection (33% versus 15%, p = 0.0003). For CRE-colonized patients the 30-day mortality was 31% and reached 36% (n = 13) at 90-days. Ceph-R-colonized patients had 27% and 33% 30- and 90-day mortality rates, respectively.

**Fig 2:**
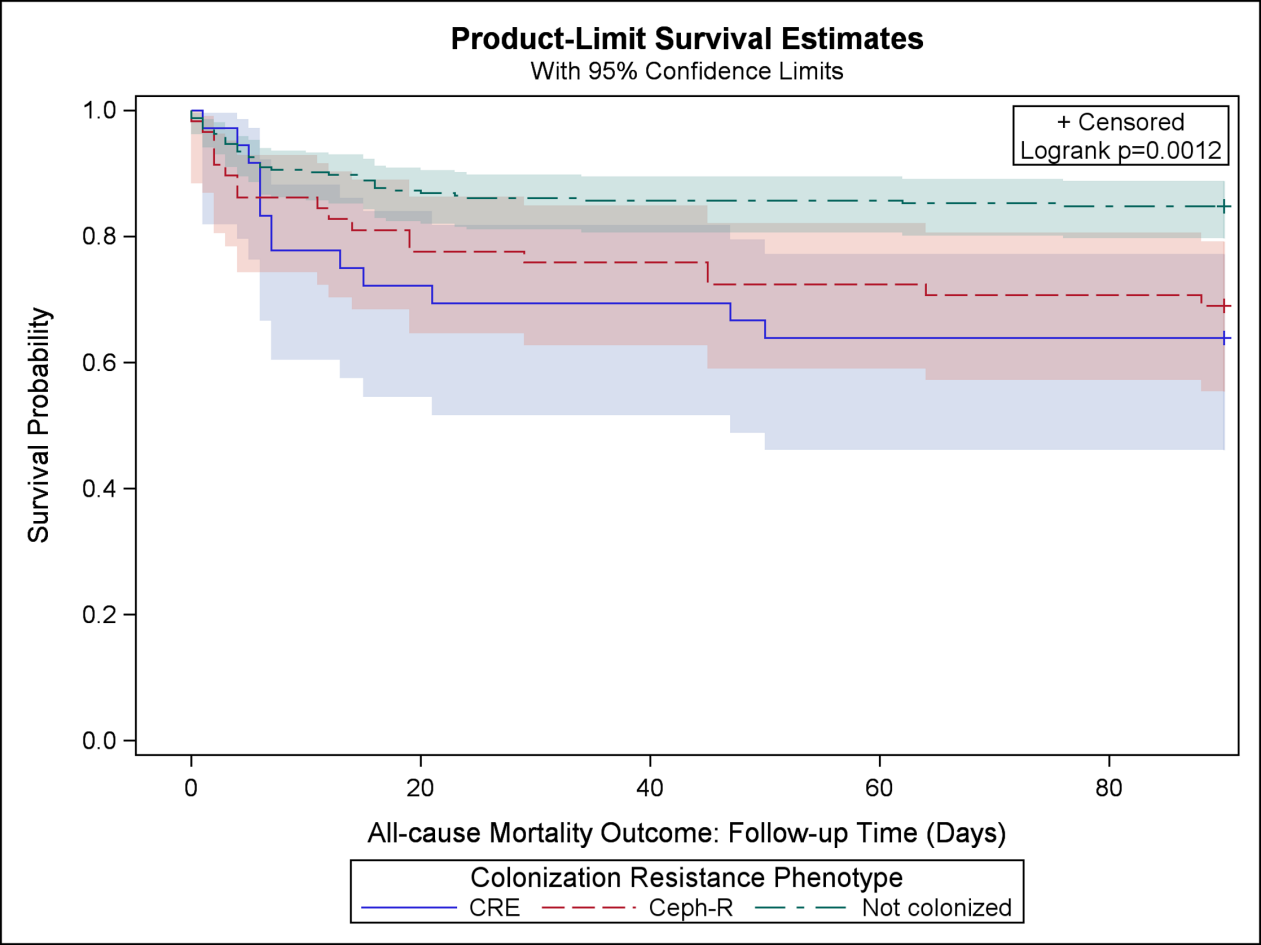
Kaplan-Meier estimates (and 95% confidence limits) of the survival probability for all-cause mortality, 90 day follow up.

In univariable analysis, there was a significant difference in mortality among CRE-colonized (n = 13, 36%), Ceph-R-colonized (n = 18, 31%), and non-colonized patients (n = 37, 15%; overall p = 0.001; Table 3). The Kaplan-Meier survival estimates also support this finding, where the Ceph-R and CRE colonized groups had lower survival probabilities than the non-colonized group (Fig 2, log-rank p = 0.001). In addition, initial admission to the MICU, renal disease, CCIS, admission diagnosis, previous Ceph-R or CRE infection, hospitalization and antibiotic use in the previous 6 months were significantly associated with 90-day mortality (Table 3).

**Table 3:** Predictors of all-cause mortality within 90 days of intensive care unit admission.

Variable n (%)	Survivor (n = 270)	Non-survivor (n = 68)	Univariable p-value	Adjusted OR (95% CI)	Multivariable p-value
**Male sex**	147 (54%)	37 (54%)	1.0	0.9 (0.5, 1.6)	0.7
**Age > 65**	127 (47%)	35 (51%)	0.5	1.2 (0.7, 2.1)	0.6
**Admitted From**			0.003		
Home	226 (84%)	45 (66%)			
SNF or Group Home	28 (10%)	17 (25%)			
OSH	16 (6%)	6 (9%)			
**ICU Type**			0.0005		0.004
Surgical	94 (35%)	9 (13%)		REF	
Medical	176 (65%)	59 (87%)		3.2 (1.4, 6.9)	
**ICU Admission Diagnosis**			0.0002		
Surgery	99 (37%)	7 (10%)			
Shock	69 (26%)	32 (47%)			
Respiratory Failure	61 (23%)	18 (26%)			
Metabolic Disarray	19 (7%)	7 (10%)			
Altered Mental Status	22 (8%)	4 (6%)			
**CCIS (mean, IQR)**	2.8 (1, 4)	3.6 (2, 5)	0.005	1.2 (1.0, 1.3)	0.045
Type II DM	85 (31%)	22 (32%)	0.9		
Heart Disease	24 (9%)	6 (9%)	1.0		
Pulmonary Disease	78 (29%)	21 (31%)	0.8		
Liver Disease	44 (16%)	14 (21%)	0.4		
Malignancy	63 (23%)	18 (26%)	0.6		
Renal Disease	47 (17%)	20 (29%)	0.03		
**Previous SOT**	39 (14%)	5 (7%)	0.1		
**Within the previous 6 months**					
**Hospitalization**	123 (46%)	52 (76%)	<0.0001	2.3 (1.2, 4.4)	0.02
**Endoscopies**					
EGD or colonoscopy	40 (15%)	13 (19%)	0.4		
ERCP or EUS	7 (3%)	1 (1%)	1.0		
**Antibiotic use**	204 (76%)	62 (91%)	0.005	1.9 (0.7, 5.9)	0.2
**Previous Ceph-R- or CRE infection**			0.0009		
No	234 (87%)	46 (68%)			
Previous Ceph-R Infection	21 (8%)	14 (21%)			
Previous CRE infection	15 (6%)	8 (12%)			
**Colonization Resistance Phenotype**			0.001		0.08
Non-colonized	207 (77%)	37 (54%)		REF	
Ceph-R colonized	40 (15%)	18 (26%)		1.8 (0.9, 3.7)	0.09
CRE colonized	23 (9%)	13 (19%)		2.3 (1.0, 5.3)	0.056

Abbreviations: OR, odds ratio; SNF, skilled nursing facility; ICU, intensive care unit; CCIS, Charlson Comorbidity Index score; IQR, intraquartile range; DM, diabetes mellitus; SOT, solid organ transplant; EGD, esophagogastroduodenoscopy; ERCP, endoscopic retrograde cholangiopancreatography; EUS, endoscopic ultrasound; Ceph-R, 3^rd^/4^th^ generation cephalosporin-resistant Enterobacteriaceae.

In a mediation analysis, we compared a simple logistic regression model containing only Ceph-R or CRE colonization to a model also containing subsequent CRE infection within 30 days of rectal swab. In the simple model, both Ceph-R and CRE colonization were significantly associated with 90-day mortality (OR 2.5, 95% CI 1.3–4.9; OR 3.2, 95% CI 1.5–6.8, respectively; p = 0.001). After the addition of subsequent CRE infection, only Ceph-R colonization remained significantly associated with 90-day mortality (Ceph-R aOR 2.5, 95% CI 1.3–4.9; CRE aOR 1.6, 95% CI 0.6–4.2; p = 0.02). Subsequent CRE infection mediated the relationship of colonization to 90-day mortality, but only for those individuals colonized with CRE organisms.

After adjusting for age, gender, CCIS score, ICU type, previous hospitalization, and antibiotic use within the previous 6 months, Ceph-R and/or CRE colonization was not a significant predictor of 90-day mortality (p = 0.08), although CRE colonization showed a trend towards increase mortality (aOR 2.3, 95% CI 1.0–5.3; p = 0.056). Admission to the MICU (aOR 3.2, 95% CI 1.4–6.9, p = 0.004), CCIS score (aOR 1.2, 95% CI 1.0–1.3, p = 0.045), and hospitalization in the past 6 months (aOR 2.3, 95% CI 1.2–4.4, p = 0.02) independently predicted 90-day mortality in the multivariable analysis.

## Discussion

Rectal colonization with CRE has previously been identified as an important epidemiological risk factor for the development of subsequent CRE infection. In a recent meta-analysis, colonized patients had a 16.5% cumulative infection rate [6]. However, these studies focused on a variety of clinical settings, were conducted during outbreaks and only few have focused on ICU patients, limiting generalizability [4, 5, 14, 25–29]. In our cohort of ICU patients, we also found CRE colonization to be a strong predictor of infection within 30 days of rectal swab collection. Here, close to 50% of our CRE-colonized patients developed a CRE infection within 30 days, representing a 10.8-fold increase in the odds of infection compared to non-colonized patients.

Notably, among patients colonized with CRE who went on to develop a CRE infection, the colonizing and infecting organism were the same species in all but one patient. This may have important implications for empiric antibiotic selection in colonized patients. Considering that the most common infection was pneumonia, aspiration of gastrointestinal contents may be a potential mechanism linking intestinal colonization with the development of infection in this critically ill cohort. Previous endoscopy or colonoscopy were also a predictor for CRE infection in the current study. Interestingly, while there have been reported outbreaks of both Ceph-R producing bacteria and CRE related to ERCP (attributed to difficulty sterilizing endoscopes) [30, 31], previous ERCP or EUS were not predictors of colonization in our cohort.

Intriguingly, Ceph-R colonization was not significantly associated with subsequent CRE infection at 30 days despite having nearly identical epidemiological risk factors [21]. The overlap in risk factors between Ceph-R and CRE colonized ICU patients also suggests that the factors contributing to CRE colonization are by themselves insufficient to produce CRE infections, and that antecedent colonization is necessary. While not significant in the multivariable model, we noted a potential inverse relationship between Ceph-R colonization and CRE infection. This may suggest that Ceph-R organisms share a colonization niche in the gut with CRE and that Ceph-R colonization could confer a degree of protection from CRE colonization and subsequent infection. The host and pathogen factors that ultimately lead to the transition from colonization to systemic infection have not been well defined.

Adverse outcomes following colonization with CRE are incompletely reported [6]. In a pooled analysis from three studies, mortality reached 10% for colonized or infected patients [6], but was much higher in patients who developed infection (30–75%). In a secondary analysis, we examined risk factors for 90-day mortality. In the multivariable analysis, only admission to the medical ICU, CCIS score, and previous hospitalization predicted 90-day mortality. Interestingly, the association between Ceph-R or CRE colonization and mortality seen in the univariate analysis was not seen in the multivariable analysis. Our study may have been underpowered to detect a significant difference, particularly given the many potential factors contributing to mortality in this ICU population.

This study had several limitations. First, in our patient population, colonization with either CRE or Ceph-R approached 30%, and our findings might not be directly translatable to areas with lower prevalence of Gram-negative drug resistance. Second, while 338 subjects were included, the cohort yielded only 26 CRE infections within the first 30 days, which limited the power of our statistical analyses. However, because the study was conducted during a 3-month period where all patients admitted to adult ICUs were screened for intestinal colonization, selection bias was limited. Third, rectal swabs were only obtained at the time of ICU admission. We were therefore unable to assess the impact of CRE acquisition or loss of colonization during the ICU stay and a number of patients may have been misclassified. Fourth, we were also unable to perform molecular typing of the isolates or to investigate bacterial factors mediating transition from colonization to infection. Fifth, we did not have access to colonizing or infecting isolates. Thus, we were we were unable to clarify the mechanism for resistance in the CRE and Ceph-R isolates (i.e. presence of carbapenemase or ESBL genes). Additionally, we had to rely on VITEK MIC data for these isolates, which can be unreliable at times. Future work is needed to identify virulence factors unique to colonizing versus infecting isolates and to identify modifiable clinical factors, e.g. use of broad-spectrum antibiotics and immunosuppressants, that may accelerate the transition from colonization to infection. Lastly, our secondary analysis was limited to all-cause, rather than attributable, mortality. Thus, we are unable to determine whether the high mortality rates seen in the CRE-infected group were directly attributable to their infection or were more likely to occur in patients with other fatal illnesses. Similarly, when analyzing mortality data, we did not consider antibiotic management or other disease management considerations, which also may have impacted outcomes.

Previous studies demonstrate the utility of rectal screening for CRE in outbreak settings to enhance identification of patients for cohorting and other infection control measures. More recently, several studies show improvement in outcomes following decolonization therapy. In a recent randomized control trial by Machuca et al., subjects colonized with carbapenem-resistant *K*. *pneumoniae* underwent decolonization therapy with an oral aminoglycoside (gentamicin or neomycin/streptomycin) [32]. At 180-day follow-up, those who underwent decolonization therapy had significantly lower mortality rates (HR 0.18) and rates of systemic infection (HR 0.15) than those who did not. A similar study demonstrated success with the use of oral polymyxin B [32]. However, it will be critical to examine decolonization therapy on a larger scale with particular attention to adverse outcomes, especially the emergence of further resistance in the targeted organisms. Fecal microbial transplantation might evolve as an alternative, less toxic strategy to restore a healthy intestinal microbiome to eliminate CRE colonization [33].

## Conclusions

Taken together, we found that in critically ill patients, CRE colonization was associated with a high risk of subsequent CRE infection and produced high mortality rates. This association was not seen in Ceph-R-colonized patients. Our findings suggest that in CRE endemic areas this screening approach may identify patients at high risk for CRE infection early and lead to early optimization of antimicrobial treatment. Future studies are needed to test novel decolonization strategies such as novel antimicrobial combinations or fecal microbial transplantation to decrease the risk of infections and poor outcomes in these critically ill patients.

## Supporting information

S1 Fig Colonization supplementary data. (XLSX)
